# Supplementary material for: Further Development of SAMPDI-3D: A Machine Learning Method for Predicting Binding Free Energy Changes Caused by Mutations in Either Protein or DNA
Source: Genes (Basel). 2025 Jan 19;16(1):101. doi: 10.3390/genes16010101 (PMC11764785; doi:10.3390/genes16010101)
Supplement: Supplementary file 1 [file genes-16-00101-s001.zip › supplimentary-figures.pdf]

Supplementary Figures

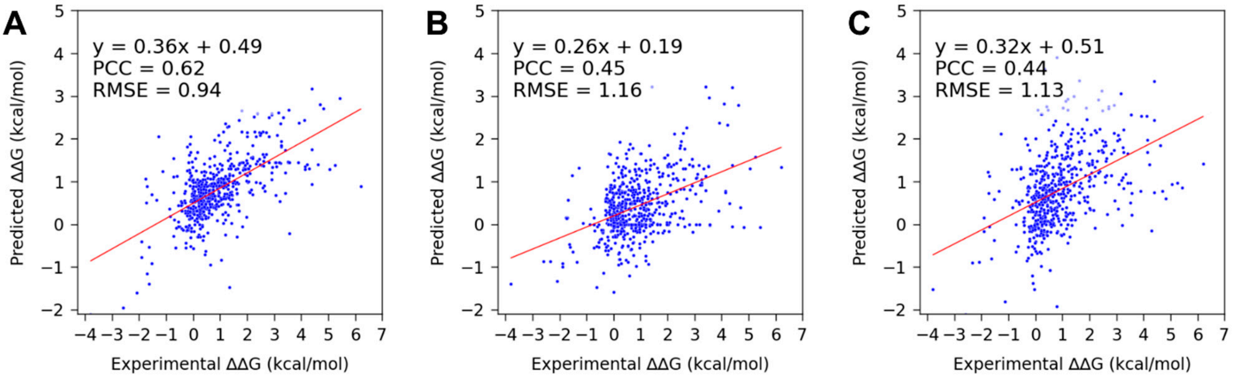

Figure S1. A plot of experimental vs. predicted binding free energy values and the fitted trend line for the complete (S596) protein mutation dataset. The predicted binding free energy was obtained using the following methods: (A) SAMPDI-3D, (B) mCSM-NA, and (C) PremPDI. The SAMPDI-3D and mCSM-NA predictions succeeded for all 596 data points in the dataset. However, PremPDI failed to predict the binding free energy change for twenty-three data points (approximately 4%).

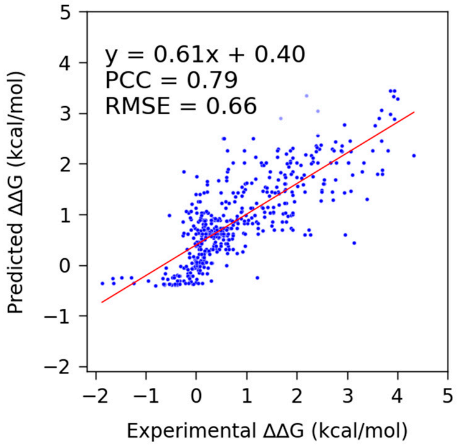

Figure S2. A plot of experimental vs. predicted binding free energy (using SAMPDI-3D) values and the fitted trend line for the complete (D502) DNA mutation dataset.
